# Examining Elastic Band Properties for Exercise Prescription: AGUEDA Equations

**DOI:** 10.1002/pri.70010

**Published:** 2024-11-27

**Authors:** Beatriz Fernandez‐Gamez, Álvaro Pulido‐Muñoz, Marcos Olvera‐Rojas, Patricio Solis‐Urra, Juan Corral‐Pérez, Javier S. Morales, David Jiménez‐Pavón, Jose Mora‐Gonzalez, Irene Esteban‐Cornejo

**Affiliations:** ^1^ Department of Physical Education and Sports Faculty of Sport Sciences Sport and Health University Research Institute (iMUDS) University of Granada Granada Spain; ^2^ Instituto de Energía Solar Universidad Politécnica de Madrid Madrid Spain; ^3^ AdventHealth Research Institute Neuroscience Institute Orlando FL USA; ^4^ Faculty of Education and Social Sciences Universidad Andres Bello Viña del Mar Chile; ^5^ Department of Physical Education ExPhy Research Group Instituto de Investigacióne Innovación Biomédica de Cádiz (INiBICA) Universidad de Cádiz Puerto Real Spain; ^6^ Department of Physical Education Faculty of Education Sciences MOVE‐IT Research Group University of Cadiz Cadiz Spain; ^7^ Biomedical Research and Innovation Institute of Cádiz (INiBICA) Research Unit, University of Cádiz Cadiz Spain; ^8^ CIBER of Frailty and Healthy Aging (CIBERFES) Instituto de Salud Carlos III Madrid Spain; ^9^ Centro de Investigación Biomédica en Red Fisiopatología de la Obesidad y Nutrición (CIBERobn) Instituto de Salud Carlos III Madrid Spain; ^10^ Instituto de Investigación Biosanitaria ibs.GRANADA Granada Spain

**Keywords:** elastic constants, personalized training, physical exercise, resistance exercise, strength training

## Abstract

**Objective:**

The objective of this paper is to quantify muscle load using Theraband elastic bands across seven resistance intensities.

**Methods:**

Bands were profiled using a force sensor, standardized to 200 cm length, and manually stretched. Measurements for each band were twice recorded at 11 distances and converted to percentages for standardization.

**Results:**

Equations derived from Theraband resistance properties were established (kg = ((elastic constant) ± SE) × percentage of elongation). The standard error ranged from 0.0007 to 0.069, while the coefficient of determination ranged from 0.977 to 0.995.

**Conclusion:**

This study provides quantification of resistance across various intensities of Theraband elastic band, offering equations for estimating external load.

## 1 Introduction

The widespread use of elastic bands as a cost‐effective and versatile tool for enhancing muscle strength (De Oliveira et al. 2017; Yoon et al. 2017; Martins et al. 2015) and function (De Oliveira et al. 2017; Sanchez‐Lastra et al. 2022; Stojanović et al. 2021; Su et al. 2022) in older adults has raised in popularity. Despite their ubiquity in exercise programs (Yoon et al. 2017; Sanchez‐Lastra et al. 2022; Davis et al. 2022; Choi, Hurr, and Kim 2020), a limited number of studies have comprehensively outlined the specific external load of elastic bands, underlining the challenge of exercise load standardization due to various characteristics, including type, elongation, resistance, or percentage of use (Page et al. 2000; Mcmaster, Cronin, and McGuigan 2010; Canfield 1999; Patterson et al. 2001; Thomas, Müller, and Busse 2005; Santos et al. 2009). This, in turn, hinders the reproducibility of protocols and the description of physical exertion, thereby introducing challenges to comparing the effects of resistance training across interventions. Previous studies have attempted to establish reference values for the tension produced by Theraband elastic bands across different color‐coded resistances (Page et al. 2000; Santos et al. 2009; Moraes Santos et al. 2009; Uchida et al. 2016; Martins et al. 2014). Uchida et al. (2016) identified significant discrepancies between the tension values provided by the manufacturer and those observed experimentally in their own study, highlighting the potential for overestimation of resistance levels. Their work underscored the importance of accurately assessing the tension generated by elastic bands to ensure safe and effective exercise prescription, especially for physical activity and rehabilitation purposes.

Understanding the mechanical properties of elastic bands is paramount for their effective use, particularly, the relationship between displacement and force. Thus, our study aims to determine the elastic constant for each elastic band hardness and provide equations to estimate external load, enabling reliable comparisons of training loads between interventions. This determination enables precise quantification of the mechanism of action based on the elastic properties of the bands, allowing for customizable intensity levels that can be tailored to individual needs and therapeutic requirements.

## 2 Methods

### 2.1 Instruments

Theraband elastic bands (Hygenic Corp.) were used in the present study (American Physical Therapy Association 2017), and have been previously employed in our randomized controlled trial, active gains using exercise during aging (AGUEDA) (Fernandez‐Gamez et al. 2023; Solis‐Urra et al. 2023). These bands are available in 7 different colors corresponding to different resistances (i.e., yellow soft, red medium, green strong, blue extra strong, black strong special, silver athletic, and gold Olympic). All the bands are made of a natural rubber latex with a length of 200 cm.

**FIGURE 1:**
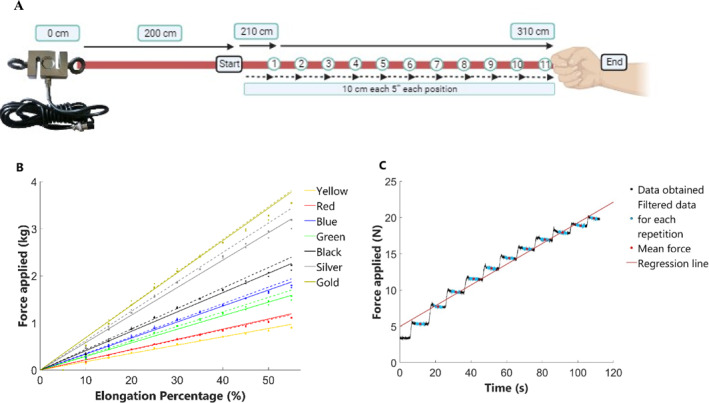
(A) Experimental condition for resistance profiling of the Theraband elastic bands. (B) Force production of the Theraband elastic bands: continuous and dashed lines represent the first and second measurement of each band, respectively. (C) Graphical representation of the repetitions of green Theraband elastic band.

We quantified the resistance force of the 7 Theraband elastic bands based on previous research protocols that have determined reference values of elastic bands (Uchida et al. 2016). A resistance test was performed by stretching the elastic bands in order to determine the relationship between force and elongation. We used an isoinertial dynamometer (Chronojump Boscosystem, Barcelona, Spain) (Erez‐Castilla et al. 2019) that consists of a cable‐extension linear position transducer attached to the barbell interfaced with a personal computer at a sampling rate of 1000 Hz. The isoinertial dynamometer was calibrated using a standardized weight of 1.25 kg before performing measurements. Then, one extremes of the elastic bands were fixed to a dynamometer, which gauged the level of resistance engendered by the band. Simultaneously, the other extremes were fastened by an evaluator using a single hand (Figure 1A). The band was securely anchored to a table to prevent slipping, allowing it to be stretched smoothly and steadily with the evaluator's entire hand. This approach ensured uniform tension and consistent force application across all tests, following protocols from previous studies to ensure methodological consistency. The magnitude of resistance was logged utilizing freely accessible open‐source software (Chronojump software 2.2.1 Db version: 2.35), and measurements were taken every 12 m with a force sensor.

### 2.2 Procedure

The elastic band was manually stretched at a consistent rate of 10 cm over every 5‐s interval. We documented the measurements from the force sensor for 11 discrete distances, starting at 200 cm (which represents the length of the band with zero elongation) and extending to accumulate a distance of 310 cm. This procedure was executed twice for each elastic band to ensure consistency of results. Data points within the initial 0–5‐s span corresponded to the unstrained state of the band. We converted the reference resistance values provided by the Chronojump software from Newton to kg to facilitate comparisons and interpretations.

Within the span of elongation to stretch the band, its behavior resembles that of a spring, exhibiting increased resistance as it moves further away from its equilibrium position. This characteristic is crucial as it facilitates the calculation of the necessary force to achieve a specific displacement of any elastic band. The initial equation used is based on Hooke's law (*F* = *k · x*) (Menezes de Souza Lima, Venceslau, and Nunes 2002), which describes the relationship between the force (*F*) applied to a spring and its displacement (*x*) from its equilibrium position (Menezes de Souza Lima, Venceslau, and Nunes 2002). Here, *k* represents the spring's elastic constant, a fundamental parameter that determines the relationship between displacement and force. The higher the *k* value the higher the force (*F*) is required for a given displacement (*x*).

To eliminate dependencies on the initial length and ensure direct comparability with Theraband data, we standardized the variable *x* by expressing it as a percentage of elongation (*P*) where *l* represents the total length of the band, and “l0” denotes the unstretched length at rest. This transformation, based on Newton's second law, is expressed as *P* (%) = (*l* − l0)/l0 × 100.

### 2.3 Statistical Analysis

Pearson correlations were performed between test and retest measurements using their mean values. Considering the correlation between both measurements were > 0.99, we averaged both measures for further analyses. Employing the mean values corresponding to each distance (specifically, 11 distances), we conducted a linear regression analysis and the least squares method to determine the spring's elastic constant of each band at various stretching distances. The elastic constant is represented by the slope of the resulting straight line. The validity of considering our elastic band as a spring and applying spring equations was assessed using the coefficient of determination (*R* ^2^), where a value close to one indicates a stronger theoretical approximation to real behavior. Data analysis was performed using MATLAB (The MathWorks, Inc. MATLAB. Version 2020a), and figures were created with BioRender.com.

## 3 Results

Table 1 presents linear regression equations for each elastic color band, derived from the mean of the two measurements conducted and referred to as “Agueda elastic constant.” This facilitates the calculation of kg corresponding to each band's percentage of elongation (i.e., (*F* = *k* · *x*): kg = ((elastic constant) ± SE) × percentage of elongation), alongside with the standard errors (i.e., SE) and the *R* ^2^ of each color. The SE ranges from 0.0007 to 0.069. The error calculation (SE) has been performed using a 2*σ* error (or standard deviation), ensuring the true value is within our margin of error with 95% confidence. The *R* ^2^ ranges from 0.977 to 0.995. Additionally, the reference values provided by Theraband (i.e., Theraband reference elastic constant) are also presented in Table 1 to facilitate direct comparisons between previous and present calculated values.

**TABLE 1:** Elastic constants for Theraband elastic bands.

Band color	Elastic constant	SE*	*R* ^2^*	r* between AGUEDA measurements
Yellow band				
AGUEDA* elastic constant	kg = 0.017 · *P*	0.0007	0.981	0.9999
Theraband reference elastic constant^a^	kg = 0.009 · *P*	0.0003	0.995	
Red band				
AGUEDA elastic constant	kg = 0.021 · *P*	0.0007	0.988	0.9997
Theraband reference elastic constant	kg = 0.011 · *P*	0.0004	0.986	
Green band				
AGUEDA elastic constant	kg = 0.030 · *P*	0.0013	0.979	0.9994
Theraband reference elastic constant	kg = 0.015 · *P*	0.0004	0.994	
Blue band				
AGUEDA elastic constant	kg = 0.035 · *P*	0.0015	0.979	0.9991
Theraband reference elastic constant	kg = 0.020 · *P*	0.0006	0.993	
Black band				
AGUEDA elastic constant	kg = 0.042 · *P*	0.0018	0.977	0.9988
Theraband reference elastic constant	kg = 0.027 · *P*	0.0011	0.986	
Silver band				
AGUEDA elastic constant	kg = 0.060 · *P*	0.0024	0.979	0.9986
Theraband reference elastic constant	kg = 0.039 · *P*	0.0011	0.993	
Gold band				
AGUEDA elastic constant	kg = 0.069 · *P*	0.0030	0.978	0.9989
Theraband reference elastic constant	kg = 0.061 · *P*	0.0020	0.989	

*Note:* Band colors indicate varying intensities, ranging from yellow (representing the lowest intensity) to gold (indicating the highest intensity). The initial equation, *F* = *k* · *x*, is represented as kg = ((elastic constant) ± SE) × percentage of elongation, where kg is the resultant resistance. For instance, when elongation is at 100%, the kg value for the yellow band would be calculated as kg = (0.00017 ± 0.0007) × 100.

Abbreviations: AGUEDA, active gains using exercise during aging; *r*, coefficient of correlation between the two AGUEDA measurements; *R* ^2^, coefficient of determination; SE, standard error.

^a^ Reference values for Theraband elastic bands' resistance across different colors (adapted from Uchida et al. (2016)).

Figure 1B represents the elastic constant obtained for each band in both repetitions. The correlation coefficients between measurements were also calculated, obtained as > 0.99 for all pairs. Figure 1C graphically shows the changes in positions every 5 s and how it causes the abrupt increases in the resistance measured by the force sensor for each elastic band. The plot illustrates changes in position over time, with the evaluator applying constant forces within the 10–15 s range at different distances and repetitions. The changes of variables have already been made to obtain the results of the constants in kg/% elongation.

## 4 Discussion

The current study presented simple equations based on elastic band's resistance properties allowing quantification of external load for muscular strength, in kilograms, using Theraband elastic bands of different intensities (colors). These findings provide an easy tool for researchers and practitioners to calculate the resistance in kilograms for each band.

Previous studies have conducted mechanical tests to identify resistance and mechanical behavior of elastic materials, including variations in elastic length (ranging from 7 cm (Martins et al. 2014) to 200 cm (Thomas, Müller, and Busse 2005)), type of elastic bands (such as tubes, loops, or plane classic elastic bands), or manufacturers (e.g., Theraband (Page et al. 2000; Uchida et al. 2016) and Elastos, Brazil (Martins et al. 2014)). Most of these studies have used the same bands as that of ours; the reference manufactures Theraband (Page et al. 2000; Thomas, Müller, and Busse 2005; Uchida et al. 2016), which are known for providing better elasticity and lower susceptibility to rupture for the bands (American Physical Therapy Association 2017). Nonetheless, researchers have identified that resistance force values of Theraband provided by the manufacturer are overestimated (Uchida et al. 2016). However, our study found that those values provided by Theraband are underestimated for all the intensities. Therefore, further research conducting mechanical tests to validate the resistance and mechanical behavior of the different Theraband elastic bands is needed.

When an elastic material is stretched, the amount of resistance in the material is proportional to the deformation of its initial length (Uchida et al. 2016), as represented by a positive slope in a linear regression analysis in all the elastic bands. The mean resistance force values obtained in our study, as well as the positive slopes in our linear regression analysis, confirm this relationship. Furthermore, the elastic constants provide valuable information about the resistance force of the bands and their behavior when stretched, which can contribute to a better quantification of external training load. This experimental method and the results obtained can help in the design and description of training loads in exercise interventions including elastic bands. To note, additional factors (e.g., individual fitness levels, specific goals, or medical conditions), and consultation with professionals trainers for optimal and safe exercise training processes, should always be considered in exercise planning.

Some limitations should be acknowledged. Firstly, our research focused exclusively on Theraband elastic bands, which limits the generalizability of our findings to other types or brands of elastic bands. Furthermore, our study design did not allow for longitudinal assessment of changes in resistance over time, which could provide valuable insights into the durability of elastic bands in terms of their elastic properties. Despite these limitations, this study lays the groundwork for future research to further explore these concepts in diverse populations and settings.

Our study provides valuable insights into the quantification of resistance load using Theraband elastic bands, offering standardized and easily implementable equations for estimating external load across different color intensities. These findings facilitate future research in quantifying resistance load and aid in the prescription of specific loading intensities for exercise interventions, ultimately enhancing the effectiveness and progression of exercise programs utilizing elastic bands.

## 5 Implications of Physiotherapy Practice

- These valuable AGUEDA equations enable the precise measurement of resistance load and the determination of loading intensities for exercise interventions using elastic bands, allowing for tailored approaches to meet individual needs and goals.
- The standardization of equations significantly enhances the precision and effectiveness of exercise programs, thereby improving the accuracy and success of physiotherapy interventions employing elastic bands.

## Conflicts of Interest

The authors declare no conflicts of interest.

## Data Availability

Research data are available upon reasonable request.
